# Vinegar-Baked Radix Bupleuri Regulates Lipid Disorders via a Pathway Dependent on Peroxisome-Proliferator-Activated Receptor-**α** in High-Fat-Diet-Induced Obese Rats

**DOI:** 10.1155/2012/827278

**Published:** 2011-12-27

**Authors:** Thing-Fong Tzeng, Hung-Jen Lu, Shorong-Shii Liou, Chia Ju Chang, I-Min Liu

**Affiliations:** ^1^Department of Internal Medicine, Pao Chien Hospital, Pingtung City, Pingtung County 90065, Taiwan; ^2^Traditional Medicinal Center, Kaohsiung Veterans General Hospital, Kaohsiung City 81362, Taiwan; ^3^Department of Pharmacy & Graduate Institute of Pharmaceutical Technology, Tajen University, Yanpu Shiang, Pingtung Shien 90701, Taiwan; ^4^School of Chinese Pharmaceutical Sciences and Chinese Medicine Resources, China Medical University, Taichung 40402, Taiwan

## Abstract

The aim of this study was to investigate the antiobesity and antihyperlipidemic effects of vinegar-baked Radix Bupleuri (VBRB) on high-fat diet- (HFD-) induced obese rats. After being fed HFD for two weeks, rats were dosed orally with VBRB or fenofibrate, once daily for further twelve weeks. VBRB (1.0 g kg^−1^ per day) produced effects similar to fenofibrate (100 mg kg^−1^) in reducing body weight (BW) gain, visceral fat-pad weights, plasma lipid levels, as well as hepatic TG and cholesterol content of HFD-fed rats. VBRB also lowered hepatic lipid droplet accumulation and the size of epididymal adipocytes in HFD-fed rats. VBRB and fenofibrate reversed the HFD-induced downregulation of hepatic peroxisome proliferator-activated receptor (PPAR)*α*. HFD-induced reductions in the hepatic levels of acyl-CoA oxidase (ACO) and cytochrome P450 isoform 4A1 (CYP4A1) proteins were reversed by VBRB and fenofibrate. The elevated expression of hepatic sterol regulatory element binding proteins (SREBPs) in HFD-fed rats was lowered by VBRB and fenofibrate. The results of this study show that VBRB suppresses BW gain and body fat accumulation by increasing fatty acid oxidation, an effect which is likely mediated via upregulation of PPAR*α* and downregulation of SREBP expression in the liver of HFD-fed rats.

## 1. Introduction

Obesity is a common chronic disorder of carbohydrate and fat metabolism, characterized by an excessive fat deposition in adipose tissue and other internal organs, such as liver, heart, skeletal muscle, and pancreatic islets [1]. Obesity remains a major global public health issue because of its increasing prevalence, which cuts across issues of sex, age group, ethnicity, or race [1]. Obesity alone can induce all the symptoms of metabolic syndrome, which is associated with many additional health problems, including increased risk of insulin resistance, nonalcoholic fatty liver, atherosclerosis, degenerative disorders such as dementia, some immune-mediated disorders such as asthma, and certain cancers [2, 3]. Pharmacological approaches to weight control have become an overriding priority [4]. Current trends for obesity management involve multiple pharmacological strategies, including blocking nutrient absorption, modulating fat metabolism, regulating adipose signals, and modulating the satiety center. However, these approaches have been associated with several serious adverse effects in the clinic, including adverse gastrointestinal effects and significant unfavorable cardiovascular effects [4]. As a result, a much safer therapeutic approach is necessary.

Radix Bupleuri, with a Chinese name Chaihu, is recorded as the roots of *Bupleurum chinense* and *B. scorzonerifolium* (family Umbelliferae) in the Chinese Pharmacopoeia, which has been widely practiced to treat influenza, fever, malaria, hepatitis, jaundice, nephritis, dizziness, bitter taste in the mouth, lung diseases, cancer, and menstrual disorders in China, Japan, and other Asian countries [5]. In previous chemical studies on Bupleurum plants, saponins, flavonoids, coumarins, fatty acids, steroids, polysaccharides, and polyacetylenes were identified [5]. Among them saponins were known to be the major bioactive compounds, which were commonly used as chemical standards for quality evaluation of Radix Bupleuri in the current Chinese Pharmacopoeia and recent publications [6]. When Radix Bupleuri was mixed thoroughly with vinegar and then baked to dry, it was changed to vinegar-baked Radix Bupleuri (VBRB). The pharmacological effect and components in the drug changed a little bit due to the vinegar-baked procedure [7]. It was demonstrated that VBRB has a much stronger effect on acesodyne and bile secretion than that of the Radix Bupleuri [8]. Therefore, VBRB was extensively used in traditional Chinese medicines for liver diseases treatment [9]. At present, although there are some reports on the pharmacological effect of VBRB, little is known about the effect of VBRB on lipid regulation besides its clinical usage.

Diet-induced obesity in rodents has been used as a model to investigate the interactions between the environment and genetics. Rats fed a high-fat diet (HFD) become obese and show distinctive visceral adiposity, dyslipidemia, hyperinsulinemia, and hepatic steatosis, which are typical of human obesity [10]. Therefore, this study investigated the effects of VBRB on body fat and lipid profiles in rats with diet-induced obesity and sought possible mechanisms of action.

## 2. Materials

### 2.1. Plant Preparations

The crude Radix Bupleuri was purchased from Jinbaoan Trade Co., Ltd. (Zhunan Township, Miaoli County 350, Taiwan) in December 2010. The identity of the plants was confirmed by Hong T.Y. (Department of Biotechnology, College of Pharmacy and Health Care, Tajen University) using macroscopic and microscopic examinations as well as thin-layered chromatography and high-performance liquid chromatography (HPLC) methods. Random amplified polymorphic DNA analysis was also applied to identify DNA polymorphisms in crude Radix Bupleuri. A voucher specimen (Lot No. 20101205) has been deposited at our laboratory. The crude Radix Bupleuri was chopped into small pieces (10 kg) and incubated with vinegar (2 kg); until vinegar was totally absorbed into Radix Bupleuri. Then, the material was dried by stir-firing to obtain VBRB.

### 2.2. Analytical Method

VBRB was ground to a powder and sieved through a 40-mesh screen. The powder was dried at 60°C until constant weight and was well-blended before use. An ASE 100 System (Dionex, Sunnyvale, CA, USA) with 34 mL stainless steel ASE vessels was used for the pressurized liquid extraction. Extraction conditions were optimized by single-factor experiments (sequentially varying the experimental parameters, one at a time, while all the other parameters remained fixed). The extract was evaporated to dryness using a rotary evaporator under 45°C. The residue was then dissolved in 5.0 mL of methanol and filtered through a 0.45 *μ*m nylon filter membrane prior to injection into the HPLC system.

An Agilent 1100 HPLC system was used, equipped with a quaternary pump, an autosampler, a degasser, an automatic thermostatic column compartment, and a DAD detector. Chromatography was performed on a TSKgel ODS-100 V C18 column (3 *μ*m, 15064.6 mm id, Tosoh, Tokyo, Japan) at a column temperature of 30°C and a flow rate of 1 mL min^−1^ using CH_3_OH (solvent A) and water (solvent B) as a mobile phase with linear gradients: 0–60 min (30–40% A), 60–80 min (40–50% A), and 80–90 min (50–100% A). DAD was set to monitor at 210 nm, and the online UV spectra were recorded in the range 190–400 nm.

For LC-ESI-MS/MS analysis, an Agilent 1100 HPLC system coupled with a LC/MSD Trap XCT mass spectrometer (Agilent Technologies, MA, USA) was used. The acquisition parameters were as follows: collision gas, ultrahighpurity helium; nebulizer gas, high purity nitrogen; nebulizer gas (N2), 35 psi; dry gas (N2), 12 L min^−1^; dry temperature, 350°C; HV voltage, 3500 V; mass range recorded mass-to-charge ratio (*m*/*z*) 200–1600, target mass *m*/*z* 800; compound stability, 100%; trap drive level, 100%; collision energy (Ampl), 0.3–2 V. Data-dependent MS/MS scanning was used in negative-ion mode so that the two most abundant ions in each MS scan were selected in turn and subjected to IT-MS (MS/MS, with MS/MS ranging from 2–4) analyses.

A 1000 *μ*g mL^−1^ standard stock solution of saikosaponin a (SSa, Sigma-Aldrich, Inc., Saint Louis, MO, USA) or saikosaponin d (SSd, Sigma-Aldrich, Inc.) was prepared and diluted in 10 *μ*g mL^−1^ to provide the working solution. To determine SSa and SSd simultaneously, SSa and SSd were used to prepare a series of standards having concentrations of 12.5, 25, 50, 100, 250, and 400 ng mL^−1^ in pure 70% methanol solution and also in the extract from VBRB to allow calibration of the samples. A calibration curve was obtained by plotting the ratio of the peak areas of the analyte and the IS as a function of the analyte concentration. A weighted (1/*x* ^2^) linear regression line was fitted over the concentration range from 12.5 to 400 ng mL^−1^. The concentrations of SSa and SSc in VBRB samples were calculated from the ratios; they are expressed in units of micrograms per gram of preparation powder.

### 2.3. Extract of VBRB

The extract of VBRB was carried out according to the method reported previously [9]. Briefly, 200 g of VBRB was socked in 2000 mL of water for 0.5 h, and then heated to boil, kept boiling for 45 min, thereafter, filtrated the extract. Gruffs were extracted again with 1600 mL of water for a further 0.5 h, pooled the filtrate, and condensed to 200 mL and then stored the extract at −20°C until use.

### 2.4. Cell Culture

3T3-L1 preadipocytes, obtained from Bioresource Collection and Research Center (BCRC 60159) of the Food Industry Research and Development Institute (Hsinchu, Taiwan), were cultured in Dulbecco's Modified Eagle's Medium (DMEM) (GIBCO BRL Life Technologies, Invitrogen Corporation, CA, USA) with 10% fetal bovine serum (FBS) (GIBCO BRL) and antibiotics (100 units mL^−1^ penicillin and 100 *μ*g mL^−1^ streptomycin). When cells were confluent, differentiation was induced by adding 0.5 mmol L^−1^ isobutylmethylxanthine (Sigma-Aldrich Co.) and 1 *μ*mol L^−1^ dexamethasone (Sigma-Aldrich Co.) to the cultures. After 2 days, cells were allowed to differentiate further by adding 10% FBS and 10 *μ*g mL^−1^ insulin (Sigma-Aldrich Co.) and the medium was changed every 2 days. At day 10, about 80% of cultures were induced to contain triglyceride (TG). Treatments including serum starvation (DMEM only), SSa, SSd, or fenofibrate (Sigma-Aldrich Co.) were given to differentiated cultures for 8 hours. All other reagents were of analytical grade.

### 2.5. Measurement of the Triglyceride Content

Oil Red O (Sigma-Aldrich Co.) at 0.2% in isopropanol (Sigma-Aldrich Co.) was mixed with water (3 : 2, v v^−1^) and filtered. Experimental cultured cells were washed with PBS, fixed by 4% paraformaldehyde (Sigma-Aldrich Co.) in PBS for 5 minutes, incubated with filtered Oil Red O for 30 minutes, and washed twice with PBS. The stained TG was extracted by isopropanol and its quantity was measured at 490 nm absorbance [11]. A total of 5 independent experiments, with triplicates each, were performed.

### 2.6. Animal Models and Treatment Protocols

Male Wistar rats, 8 weeks of age, were obtained from the National Laboratory Animal Center (Taipei, Taiwan). They were maintained in a temperature-controlled room (25 ± 1°C) on a 12 h : 12 h light-dark cycle (lights on at 06:00 h) in the animal center (Tajen University, Ping Tung Shien, Taiwan). Food and water were available *ad libitum*. Regular rat chow diet (RCD, #D12450B, Research Diets, New Brunswick, NJ) with 20 kcal% protein, 70 kcal% carbohydrate, and 10 kcal% fat from lard was used as the maintenance and control diet. A purified ingredient HFD with 20 kcal% protein, 35 kcal% carbohydrate, and 45 kcal% fat primarily from lard (#D12451, Research Diets) was used to induce a rapid increase in body weight (BW) and obesity [12]. The caloric density of the control diet was 3.85 kcal g^−1^; that of the HFD was 4.73 kcal g^−1^. All animal procedures were performed according to the Guide for the Care and Use of Laboratory Animals of the National Institutes of Health as well as the guidelines of the Animal Welfare Act. These studies were conducted with the approval of the Institutional Animal Care and Use Committee (IACUC) at Tajen University (approval number: IACUC 98-13; approval date: October 19, 2009).

After being fed a HFD for two weeks, rats were dosed by oral gavage once per day for 12 weeks with VBRB doses of 0.3, 0.5, and 1.0 g kg^−1^ in a volume of 1.5 mL kg^−1^ distilled water. Another group of HFD-fed rats was treated orally for 12 weeks with 100 mg kg^−1^ per day fenofibrate. In folk medicine, the recommended daily oral dose of VBRB is 3–10 g per adult per day. The dose of VBRB was calculated by multiplying the recommended dose of intake per kilogram by the human metabolism coefficient to get the dose of intake per day of rats according to the equation: the intake dose per kilogram of rat body weight = recommended intake dose for humans ÷ body weight 60 kg × 6.25 [13]. The approximate daily oral dose of VBRB for a rat is ranged from 0.3–1.0 g kg^−1^. The dose of fenofibrate was based on studies with long-term fenofibrate treatment in rats [14]. A vehicle-control group of HFD-fed and RCD-fed rats was treated with 1.5 mL kg^−1^ distilled water only over the same treatment period. Each experimental group contained 8 rats in the study. Twelve weeks after treatment with VBRB or fenofibrate, HFD-fed rats were weighed and anesthetized with sodium pentobarbital (30 mg kg^−1^) administered intraperitoneally (i.p.), and blood samples were collected from the lateral tail vein. Feed and water were supplied *ad libitum* throughout the 12-week experimental period. Samples were centrifuged at 2,000 ×g for 10 minutes at 4°C. The plasma was then removed and placed into aliquots for the respective analytical determinations. After blood was collected, the liver and visceral and subcutaneous white adipose tissues (WAT) were removed, rinsed with physiological saline, weighed, and immediately stored at −70°C.

### 2.7. Biochemical Parameter Analysis

Diagnostic kits for the measurement of plasma glucose (Cat. No. 10009582), total cholesterol (TC; Cat. No. 10007640), and triglycerides (TG; Cat. No. 10010303) were purchased from Cayman Chemical Company (Michigan, USA). The diagnostic kit to determine plasma and hepatic levels of high density lipoprotein cholesterol (HDL-C) was purchased from Bio-Quant Diagnostics (Cat. No. BQ 019CR, CA, USA), and low density lipoprotein cholesterol (LDL-C) levels were calculated using Friedewald's equation [15]. Plasma-free fatty acid (FFA) levels were determined using an FFA-quantification kit obtained from Abcam plc (Cat. No. ab65341, MA, USA). All samples were analyzed in triplicate. Atherogenic index (AI) and coronary risk index (CRI) were calculated as LDL-C/HDL-C and TC/HDL-C, respectively [16, 17].

### 2.8. Extraction of Hepatic Lipid

A section of each liver was collected for lipid content analysis. The liver (1.25 g) was homogenized with chloroform/methanol (1 : 2, 3.75 mL), and then chloroform (1.25 mL) and distilled water (1.25 mL) were added to the homogenate and mixed well. After centrifugation (1,500 ×g for 10 min), the lower clear organic phase solution was transferred into a new glass tube and then lyophilized. The lyophilized powders were dissolved in chloroform/methanol (1 : 2) as the hepatic lipid extracts and stored at −20°C for fewer than three days [18].

### 2.9. Hepatic Pathological Evaluation

Small pieces of hepatic tissues taken from experimental animals were fixed in 10% neutral formalin, dehydrated with alcohol, embedded in paraffin, and sectioned to a mean thickness of 4 *μ*m. Hematoxylin- and- eosin- (H&E-) stained tissues were examined histologically to evaluate the index of diabetic-induced necrosis. Liver biopsy was scored according to the following criteria: grade 0: no steatosis, normal liver; grade 1: <25% of hepatocytes affected; grade 2: 26–50% of hepatocytes affected; grade 3: 51–75% of hepatocytes affected; and grade 4: >76% of hepatocytes affected [19]. A total of 5 independent experiments were carried out for statistical analysis.

### 2.10. Adipocyte Pathological Evaluation

Histological photomicrographs of adipose tissue were analyzed with a light microscope using the paraffin method. Fresh tissues were fixed immediately in Bouin's solution for 6 to 12 hours, and then fixed tissues were washed under running water. After being dehydrated through different grades of alcohol, the tissues were embedded in paraffin block at 60°C. Sections (8 *μ*m) were cut and mounted on glass slides coated with an egg albumin, and the paraffin was removed using xylene and alcohol. The tissues were stained with H&E. After being dehydrated and cleared of alcohol and xylene, the glass slides were mounted in Canada Balsam. Photomicrographs were taken with a Zeiss Axiolab light microscope equipped with a Nikon Microflex HFX microscope camera. The sizes of epididymal adipocytes were calculated using Image-Pro Plus 7.0 (Media Cybernetics, MD, USA). A total of 5 independent experiments were carried out for statistical analysis.

### 2.11. Preparation of Hepatic Fractions

Hepatic fractions were prepared as described previously [20]. To prepare nuclear fractions, hepatic tissue was homogenized with ice-cold lysis buffer containing 5 mmol L^−1^ Tris-HCl (pH 7.5), 2 mmol L^−1^ MgCl_2_, 15 mmol L^−1^ L CaCl_2_, 1.5 mol L^−1^ sucrose, 0.1 mol L^−1^ dithiothreitol (DTT), and protease inhibitor cocktail. After centrifugation (10,500 ×g for 20 minutes at 4°C), the pellet was suspended in extraction buffer containing 20 mmol L^−1^ 2-[4-(2-hydroxyethyl)-1-piperazinyl]ethanesulfonic acid (pH 7.9), 1.5 mmol L^−1^ MgCl_2_, 0.42 mol L^−1^ NaCl, 0.2 mmol L^−1^ L EDTA, 25% (v v^−1^) glycerol, 0.1 mol L^−1^ DTT, and protease inhibitor cocktail. The mixture was placed on ice for 30 minutes. The nuclear fraction was prepared by centrifugation at 20,500 ×g for 5 minutes at 4°C. The postnuclear fraction was extracted from the liver of each rat as described below. In brief, hepatic tissue was homogenized with ice-cold lysis buffer (pH 7.4) containing 137 mmol L^−1^ NaCl, 20 mmol L^−1^ Tris-HCl, 1% Tween 20, 10% glycerol, 1 mmol L^−1^ phenylmethylsulfonyl fluoride, and protease inhibitor cocktail solution in DMSO. The homogenate was then centrifuged at 2,000 ×g for 10 minutes at 4°C. The protein concentration of each fraction was determined using a commercial kit (Bio-Rad Laboratories, Hercules, CA, USA).

### 2.12. Western Blot Analyses

For the determination of peroxisome-proliferators-activated-receptor (PPAR), sterol-regulatory-element-binding protein (SREBP)-1 and SREBP-2, 30 mg protein of each nuclear fraction was resolved using 8% sodium dodecylsulfate polyacrylamide gel electrophoresis (SDS-PAGE). Separated proteins were transferred electrophoretically to a nitrocellulose membrane, blocked with 5% (w v^−1^) skim milk solution for 1 hour, and then incubated with primary antibodies to PPAR*α* (Santa Cruz Biotechnology, Inc., CA, USA; Cat. No. sc-1985), SREBP-1 (Santa Cruz Biotechnology, Inc.; Cat. No. sc-367), SREBP-2 (Santa Cruz Biotechnology, Inc.; Cat. No. sc-5603), or *β*-actin (Santa Cruz Biotechnology, Inc.; Cat. No. sc-130656) overnight at 4°C. After the blots were washed, they were incubated with goat anti-rabbit and/or goat anti-mouse IgG HRP-conjugated secondary antibody for 1.5 hours at room temperature. The blots were stripped with Restore Western Blot Stripping Buffer (CANDOR Bioscience GmbH, Wangen, Germany) for 15 minutes and incubated with the antibodies. In addition, 30 mg protein from each postnuclear fraction for acyl-CoA oxidase (ACO; Santa Cruz Biotechnology, Inc.; Cat. No. sc-98499) and cytochrome P450 isoform 4A1 (CYP4A1; Santa Cruz Biotechnology, Inc.; Cat. No. sc-53248) was subjected to 10% SDS-PAGE. Each antigen-antibody complex was visualized using ECL Western Blotting Detection Reagents and detected by chemiluminescence with LAS-1000 plus (Fujifilm, Tokyo, Japan). Band densities were determined using ATTO Densitograph Software (ATTO Corporation, Tokyo, Japan) and quantified as the ratio to *β*-actin. The mean value for samples from the vehicle-treated RCD-fed group on each immunoblot, expressed in densitometry units, was adjusted to a value of 1.0. All experimental sample values were then expressed relative to this adjusted mean value. A total of 5 independent experiments were carried out for statistical analysis.

### 2.13. Statistical Analysis

Data are expressed as the mean ± standard deviation (SD) for each group of animals at the number (*n*) indicated in tables. Statistical analysis was performed with one-way analysis of variance (ANOVA). The Dunnett range posthoc comparisons were used to determine the source of significant differences where appropriate. A *P* value <.05 was considered statistically significant.

## 3. Results

### 3.1. Quantitative Analysis

**Figure 1:**
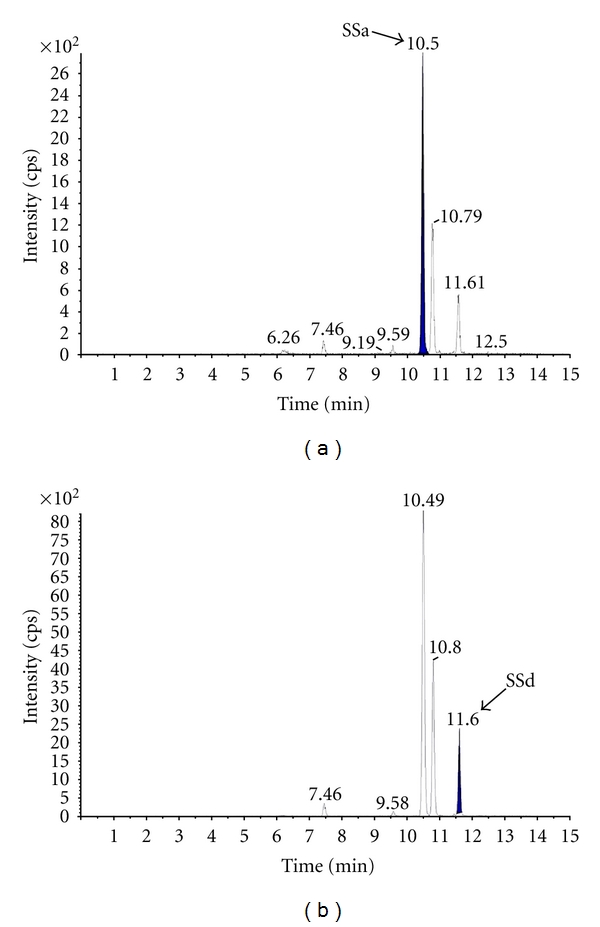
Mass chromatograms of (a) SSa and (b) SSd in VBRB extract.

The regression equations of SSa and SSd are *y* = 190.65*x* − 1219.72 (*R* ^2^ = 0.9992) and *y* = 42.87*x* − 111.25 (*R* ^2^ = 0.9983), respectively. The content of SSa (724.63 ± 0.06 *μ*g/g) was higher than that of SSd (9.62 ± 0.04 *μ*g/g) in VBRB. The chromatogram of the sample solution has been shown in Figure 1.

### 3.2. Effects on Lipogenic Differentiated 3T3-L1 Adipocyte

**Figure 2:**
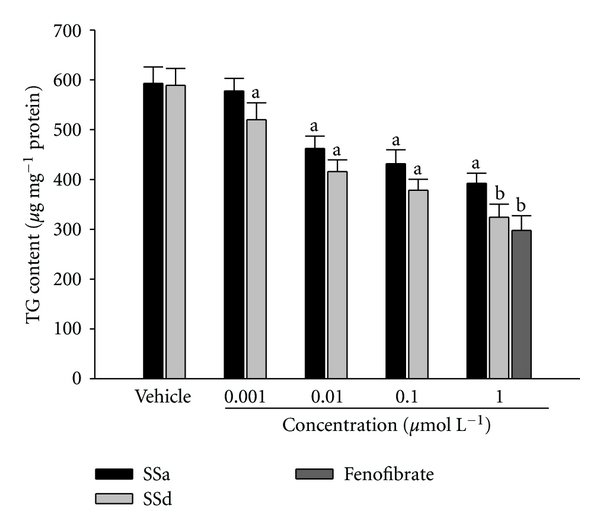
Effect of SSa, SSd, or fenofibrate on triglyceride (TG) content in 3T3-L1 adipocytes. The vehicle (distilled water) used to solve the tested medications was given at the same volume. Values were expressed as mean with SD (*n* = 5 per group) in each column. ^a^ *P* < .05 and ^b^ *P* < .01 compared to the values of vehicle-treated group.

SSa and SSd exhibited a significant concentration-dependent decrease in the intracellular accumulation of TG in 3T3-L1 adipocytes; the most significant effect (over 30% TG reduction) was observed in treatment at 1 *μ*mol L^−1^ (*n* = 5; Figure 2). Fenofibrate (1 *μ*mol L^−1^) caused a decrease in the TG content of differentiated 3T3-L1 adipocytes by 41% (*n* = 5; Figure 2).

### 3.3. Effects on Body Weight (BW) and Food Intake

As shown in Table 1, the BWs of HFD-fed rats in the drug-treated and vehicle-treated groups were monitored over the 12-week treatment period. At the end of treatment, the BW of VBRB-treated rats (*n* = 8) was significantly lower than that of rats in the vehicle-treated group (*n* = 8). VBRB significantly suppressed BW gain at both the moderate (0.5 g kg^−1^ per day; *n* = 8) and high doses (1.0 g kg^−1^ per day; *n* = 8). Similar results were seen in rats treated with fenofibrate (100 mg kg^−1^ per day; *n* = 8). No significant differences in daily food intake were observed among the groups over the experimental period, despite the slightly higher water intake observed in the vehicle-treated HFD-fed group as compared to the others (*n* = 8; Table 1).

### 3.4. Effects on Weight of White Adipose Tissue (WAT)

The weights of WAT from HFD-fed rats in the drug-treated and vehicle-treated groups were assessed after the treatment period (Table 1). Epididymal WAT, perirenal WAT, mesenteric WAT, and inguinal WAT weights were lower in VBRB-treated rats (*n* = 8) than in their vehicle-treated counterparts (*n* = 8). A significant (*P* < .05) reduction in WAT weight was seen with both the moderate (0.5 g kg^−1^ per day; *n* = 8) and high doses (1.0 g kg^−1^ per day; *n* = 8) of VBRB. The weight of epididymal, perirenal, mesenteric, and inguinal fat pads was reduced significantly by 27.5%, 29.6%, 28.5%, and 32.4%, respectively, in VBRB- (1.0 g kg^−1^ per day) treated, and HFD-fed rats compared with vehicle-treated, HFD-fed rats (Table 1). Similarly, after treatment with fenofibrate, epididymal, perirenal, mesenteric, and inguinal fat pads were 30.4%, 35.4%, 34.5%, and 36.4% lower, respectively, than in their vehicle-treated counterparts (*n* = 8, Table 1).

### 3.5. Effect on Plasma Lipids

The HFD caused elevated concentrations of plasma TC, TG, and LDL-C (Table 2). The moderate (0.5 g kg^−1^ per day) and high doses (1.0 g kg^−1^ per day) of VBRB significantly reduced plasma total TC levels (23.5% and 31.5% reduction, resp.) compared with vehicle-treated, HFD-fed rats (*n* = 8; Table 2). All doses of VBRB decreased plasma TG levels in HFD-fed rats; the high doses of VBRB reduced plasma TG levels in HFD-fed rats to 64.4% of their vehicle-treated counterparts (*n* = 8; Table 2). The low (0.3 g kg^−1^ per day), moderate, and high doses of VBRB significantly reduced plasma LDL-C levels (23.0%, 38.2%, and 43.3% reductions, resp.; Table 2). Plasma TC, TG, and LDL-C concentrations were reduced significantly by 38.4%, 47.3%, and 59.7%, respectively, in fenofibrate-treated, HFD-fed rats compared with vehicle-treated, HFD-fed rats (*n* = 8; Table 2).

The plasma concentration of HDL-C in HFD-fed rats was reduced to 59.3% of the level in the RCD-fed group (*n* = 8; Table 2). After 8 weeks of treatment with VBRB (1.0 g kg^−1^ per day; *n* = 8) or fenofibrate (*n* = 8), the plasma HDL-C concentration in HFD-fed rats was elevated to nearly that of the RCD-fed rats.

Plasma FFAs were significantly higher in vehicle-treated HFD-fed rats compared to RCD-fed rats (Table 2). The plasma FFA level was reduced by 37.4% in HFD-fed rats treated with 1.0 g/kg/day VBRB, compared with their vehicle-treated counterparts (*n* = 8; Table 2). Fenofibrate treatment reduced FFA levels in HFD-fed rats by 46.4% relative to the level in vehicle-treated, HFD-fed rats (*n* = 8 in each group; Table 2).

Fenofibrate treatment arrested the elevation of AI and CRI in HFD-fed rats (*n* = 8; Table 2). VBRB treatment also caused a significant (*P* < .05) and dose-related reduction in the atherogenic and coronary artery risk indices in the HFD-fed rats when compared to the values recorded for their vehicle-treated counterparts (*n* = 8; Table 2).

### 3.6. Effect on Hepatic Lipids

The hepatic TC level was significantly higher in HFD-fed rats than in rats from the RCD-fed group (*n* = 8; Table 2). The hepatic TC levels were reduced by 33.4% in HFD-fed rats treated with 1.0 g kg^−1^ per day VBRB (*n* = 8; Table 2). Similarly, VBRB treatment (1.0 g kg^−1^ per day) also produced a significant reduction in hepatic TG concentration, to 64.5% of that in vehicle-treated, HFD-fed rats (*n* = 8; Table 2). Hepatic TC and TG levels were significantly reduced (by 44.2% and 43.7%, resp.) in fenofibrate-treated rats compared with vehicle-treated, HFD-fed rats (*n* = 8; Table 2).

### 3.7. Morphological Changes in Hepatocytes

**Figure 3:**
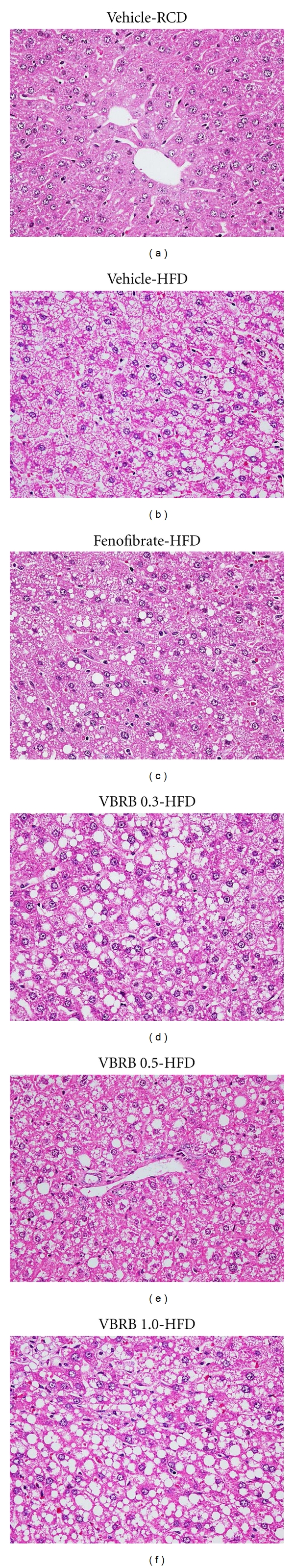
Histopathological findings in livers of HFD-fed rats receiving 12-weeks treatment with VBRB or fenofibrate. Rats not receiving any treatment were given the same volume of vehicle (distilled water) used to solve the test medications. Photomicrographs were taken at a magnification of ×400. Photomicrographs are of tissues isolated from vehicle-treated RCD-fed rats (vehicle-RCD), vehicle-treated HFD-fed rats (vehicle-HFD), VBRB- (0.3 g kg^−1^ per day) treated HFD-fed rats (VBRB 0.3-HFD), VBRB- (0.5 g kg^−1^ per day) treated HFD-fed rats (VBRB 0.5-HFD), VBRB- (1.0 g kg^−1^ per day) treated HFD-fed rats (VBRB 1.0-HFD), or fenofibrate (100 mg kg^−1^ per day)-treated HFD-fed rats (fenofibrate-HFD). The pathological grading of hepatic steatosis has been indicated in Table 3.

HFD-fed rats showed significantly greater hepatic lipid accumulation than RCD-fed animals (Figure 3). The extent of hepatic lipid accumulation after 12 weeks in fenofibrate-treated, HFD-fed rats was similar to that in RCD-fed rats (Figure 3). HFD-fed rats treated with 1.0 g kg^−1^ per day VBRB showed considerably lower hepatic lipid accumulation than their vehicle-treated counterparts (Figure 3). The pathological grading of hepatic steatosis has been indicated in Table 3 (*n* = 5).

### 3.8. Morphological Changes in Epididymal Adipocytes

**Figure 4:**
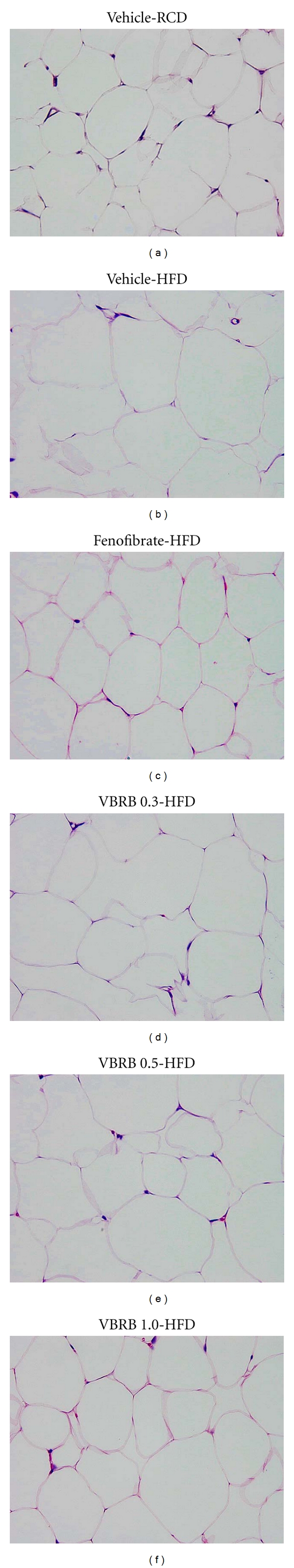
Histopathological findings in epididymal white adipose tissue of HFD-fed rats receiving 12-weeks treatment with VBRB or fenofibrate. Rats not receiving any treatment were given the same volume of vehicle (distilled water) used to solve the test medications. Photomicrographs were taken at a magnification of ×400. Photomicrographs are of tissues isolated from vehicle-treated RCD-fed rats (vehicle-RCD), vehicle-treated HFD-fed rats (vehicle-HFD), VBRB- (0.3 g kg^−1^ per day) treated HFD-fed rats (VBRB 0.3-HFD), VBRB- (0.5 g kg^−1^ per day) treated HFD-fed rats (VBRB 0.5-HFD), VBRB- (1.0 g kg^−1^ per day) treated HFD-fed rats (VBRB 1.0-HFD), or fenofibrate (100 mg kg^−1^ per day)-treated HFD-fed rats (fenofibrate-HFD). Quantification of the data is shown in Table 3.

The histological appearance of epididymal adipocytes was irregular in HFD-fed rats compared with animals in the RCD-fed group (*n* = 5; Table 2). This morphological change was not evident in HFD-fed rats after fenofibrate treatment (Figure 4). The histological appearance of epididymal adipocytes in HFD-fed rats treated with 1.0 g kg^−1^ per day VBRB was not as normal as in fenofibrate-treated rats, but was better than in vehicle-treated controls (Figure 4). Epididymal adipocytes were also significantly larger in HFD-fed compared to RCD-fed rats (Figure 4). The average size of epididymal adipocytes was reduced by approximately 36.4% and 4.3%, respectively, in HFD-fed rats treated with 1.0 g kg^−1^ per day VBRB or fenofibrate compared with their vehicle-treated counterparts (*n* = 5; Table 3).

### 3.9. Protein Expressions of PPAR*α*, ACO, CYP4A, and SREBPs in Hepatic Tissues

**Figure 5:**
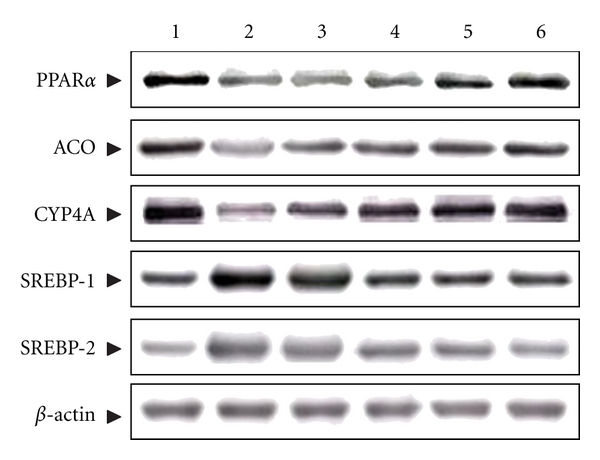
Representative immunoblots of protein expression of PPAR*α*, ACO, CYP4A, and SREBPs in hepatic tissues. Photomicrographs are of hepatic tissues isolated from vehicle-treated RCD-fed rats (line 1), vehicle-treated HFD-fed rats (line 2), VBRB- (0.3 g kg^−1^ per day) treated HFD-fed rats (line 3), VBRB- (0.5 g kg^−1^ per day) treated HFD-fed rats (line 4), VBRB- (1.0 g kg^−1^ per day) treated HFD-fed rats (line 5), or fenofibrate- (100 mg kg^−1^ per day) treated HFD-fed rats (line 6). Rats not receiving any treatment were given the same volume of vehicle (distilled water) used to solve the test medications.

Hepatic PPAR*α* protein expression in HFD-fed rats was lower than that in RCD-fed animals, but was elevated significantly by treatment with 1.0 g kg^−1^ per day VBRB (Figure 5). In addition, the hepatic expression levels of ACO and CYP4A proteins in HFD-fed rats were markedly lower than in RCD-fed rats, but were significantly elevated in VBRB- (1.0 g kg^−1^ per day) treated HFD-fed rats (Figure 5). Similar results were seen in rats treated with fenofibrate.

The expression levels of hepatic SREBP-1 and SREBP-2 proteins in HFD-fed rats were significantly higher in RCD-fed rats (Figure 5). The proteins levels of hepatic SREBP-1 and SREBP-2 were decreased by 49.3% and 53.7% in HFD-fed rats treated with VBRB (1.0 g kg^−1^ per day), relative to those in vehicle-treated, HFD-fed rats, respectively (Figure 5). Hepatic SREBP-1 and SREBP-2 protein expression levels in HFD-fed rats treated with fenofibrate were 48.1% and 43.1%, respectively, lower than those in their vehicle-treated counterparts (Figure 5).

## 4. Discussion

In the this study, BW loss in HFD-fed rats was accompanied by a depletion of body fat stores, since treatment with VBRB also significantly reduced the weight of the visceral and subcutaneous WAT compared with that of vehicle-treated HFD-fed rats. Excessive growth of adipose tissue results in obesity, which involves two growth mechanisms: hyperplasia (cell number increase) and hypertrophy (cell size increase) [21]. The histological appearance of white adipocytes in HFD-fed animals supplemented with fenofibrate or VBRB (1.0 g kg^−1^ per day) was more regular, and adipocytes were similar in size to those in RCD-fed rats. This suggests that VBRB suppresses the HFD-induced increase in adipose tissue mass and BW gain and that it may inhibit lipid accumulation in adipose tissue in particular.

Obesity, especially abdominal obesity, is associated with dyslipidemia, characterized by elevated TG and reduced HDL-C concentrations [22]. TG is involved in the ectopic accumulation of lipid stores in the liver and is associated with a number of diseases such as metabolic syndrome and type 2 diabetes [23]. High TC levels increase the risk of developing coronary heart disease, and high levels of LDL-C are a risk factor for coronary heart disease, while high HDL-C is helpful in transporting excess cholesterol to the liver for excretion in the bile [24]. As a result, HDL-C levels are inversely related to coronary heart disease risk [25]. Similar to fenofibrate treatment, the oral administration of VBRB significantly lowered plasma TC, TG, and LDL-C levels in rats with HFD-induced obesity. Thus, VBRB may be of benefit to patients with hypercholesterolemia and hypertriglyceridemia.

The effect of VBRB on the atherogenic and coronary artery risk indices is also notable. The ratio of total cholesterol to HDL-C (i.e., the atherogenic index) and the ratio of LDL-C to HDL-C (i.e., the coronary artery index) are strong and reliable indicators of whether or not cholesterol is deposited into tissues or metabolized and excreted [26]. The results of this study show that treatment with VBRB or fenofibrate causes profound reductions in the atherogenic and coronary indices in experimental hyperlipidemic rats. This strongly suggests that VBRB has therapeutic potential for the management of obesity and hyperlipidemia and for the prevention of atherogenic cardiovascular diseases.

Due to the ability of VBRB to reduce serum levels of TGs and total cholesterol, as well as adipose tissue mass and BW gain functions similar to those of PPAR*α* activation; we hypothesized that the actions of VBRB are related to the regulation of hepatic expression of PPAR*α*-target genes involved in lipid metabolism. In hepatocytes and other tissues (e.g., heart), ligand- (natural long-chain fatty acids) activated PPAR*α* binds to peroxisome proliferator response elements of DNA and increases the transcription of genes encoding enzymes involved in fatty acid oxidation and lipoprotein metabolism [27]. The outcome is an increase in hepatic fatty acid oxidation and ketogenesis, decreased lipid levels in tissues, and protection against lipotoxicity. We found that VBRB-treated HFD-fed rats had significantly higher hepatic PPAR*α* protein, and that the effect of VBRB was equivalent to that of fenofibrate. It appears that VBRB regulates the lipid profile in HFD-fed rats through an increase in hepatic PPAR*α* levels. The discovery of VBRB as a PPAR*α* activator may offer the promise of a novel class of an antidiabetic drug.

Although reductions in lipogenic activity and in dietary lipid absorption have been suggested as causes of the reduced liver lipid content, an increased capacity of peroxisomal *β*-oxidation (ACO) and microsomal *ω*-oxidation (CYP4A) of fatty acids could also be contributing factors [28]. Elevated hepatic levels of ACO and CYP4A proteins imply an enhanced oxidation of fatty acids in the peroxisomes and microsomes of VBRB-treated, HFD-fed rats. These results support the contention that VBRB, by directly or indirectly activating PPAR*α*, can upregulate the expression of genes downstream of PPAR*α*, which may lead to an enhanced hepatic fatty acid oxidation and reduced TG content.

The sterol-regulatory-element-binding proteins (SREBPs) are a family of three basic helix-loop-helix leucine zipper transcription factors (SREBP-1a, -1c, and -2) that have been identified as transacting factors involved in the maintenance of intracellular cholesterol homeostasis, the control of fatty acid metabolism, and the differentiation of adipocytes [29]. The SREBP-2 isoform activates genes of the cholesterogenic pathway, whereas the SREBP-1 isoforms are more active in regulating the synthesis of fatty acids [30]. It has been documented that SREBPs were the upregulated genes related to fatty acid synthase and cholesterol levels [20]. In the current study, the elevated expressions of SREBP-1 and SREBP-2 in HFD-fed rats were significantly decreased by the VBRB (1.0 g kg^−1^ per day) treatment. These results suggest that VBRB has an ameliorating effect on dyslipidemia through the impaired hepatic SREBPs as well.

Three major oleanane saponins named SSa, SSc, SSd, and many minor saponins are isolated from the roots of Radix Bupleuri [6]. The following activities are reported for SSa and SSd: anticancer, anti-inflammation, corticosterone secreting, plasma-cholesterol-lowering action, hemolytic activity, effects on membrane fluidity, and a protective action against hepatic damage [5]. However, no such biological activities are observed for SSc. Adipocytes play an important role in lipid homeostasis and energy balance by relating to TG storage and free fatty acids release [31]. The antiobesity effect therefore could be represented by the suppression of TG formation in 3T3-L1 adipocytes. Antiobesity effect of SSa or SSd in the 3T3-L1 cell model was measured in our study. We found that treatment with both saikosaponins reduced TG content up to 30% at the concentration of 1 *μ*mol L^−1^, which was similar to the effect produced by fenofibrate at same concentration, while the content of SSa was higher than that of SSd in VBRB. SSd seems to play a major role in the attenuation of visceral fat accumulation and improvement of hyperlipidemia produced by VBRB, providing a rationale for the use of this product in lipid disorders regulation in folk medicine.

Although we suggest that supplemental treatment with VBRB may prevent or improve obesity by modulating lipid metabolism caused by an excessive HFD, the results come from rat studies and thus cannot be generalized to human. Thus, placebo-controlled human studies are required to find the usability of this plant in human cholesterol/lipid/obesity indications due to different fat/lipid metabolism in humans and rats. Also, safety testing should be taken with the chronic consumption of large doses of traditional remedies, especially in in pregnant women, children, old people, and people with kidney diseases; the results from rat studies cannot be generalized to human.

## 5. Conclusion

The results of this study show that VBRB suppresses BW gain and body fat accumulation by increasing fatty acid oxidation, an effect which is likely mediated via the upregulation of PPAR*α* and downregulation of SREBP expression in the liver of HFD-fed rats. SSd from VBRB could account for the observed bioactivity. This experiment in rats did not give any rationale to use VBRB in humans in these conditions due to different lipoprotein/lipid metabolism in rats and humans; instead it may give rationale for controlled human studies.

## Figures and Tables

**Table 1:** Changes on the body weight (BW), food and water intake, and weight of white adipose tissue (WAT) in HFD-fed rats receiving 12-weeks treatment with VBRB or fenofibrate.

Groups	Initial BW (g rat^−1^)	BW gain (g rat^−1^)	Food intake (g rat^−1^ per day)	Water intake (mL rat^−1^ per day)	WAT (mg 100 g^−1^ BW)			
					Epididymal	Perirenal	Mesenteric	Inguinal
RCD-fed								
Vehicle-treated	172.5 ± 7.7	17.8 ± 6.2^b^	19.3 ± 5.9	77.2 ± 8.1	303.6 ± 15.8^b^	172.9 ± 12.7^b^	129.8 ± 9.6^b^	146.9 ± 11.2^b^
HFD-fed								
Vehicle-treated	173.4 ± 6.9	53.7 ± 7.0	20.3 ± 6.7	86.3 ± 9.4	422.1 ± 21.3	263.8 ± 14.2	191.7 ± 13.6	223.6 ± 14.2
VBRB (g kg^−1^ per day)								
0.3	172.3 ± 9.3	41.4 ± 6.7	20.9 ± 6.8	82.4 ±10.2	368.1 ± 16.1^a^	228.6 ± 13.5	171.5 ± 13.1	194.3 ± 11.8
0.5	173.1 ± 8.4	30.7 ± 6.9^a^	21.8 ± 6.9	80.8 ± 9.7	338.5 ± 22.8^a^	198.9 ±13.9^a^	156.2 ±10.3^a^	170.6 ± 11.0^a^
1.0	172.8 ± 6.7	23.6 ± 5.3^b^	20.3 ± 6.3	78.1 ± 7.9	308.4 ± 19.2^b^	185.3 ± 12.1^b^	136.9 ± 8.7^b^	151.1 ± 9.2^b^
Fenofibrate (100 mg kg^−1^ per day)	171.6 ± 8.1	20.4 ± 5.8^b^	19.9 ± 7.1	76.7 ± 8.9	293.9 ± 14.6^b^	170.2 ± 11.7^b^	125.6 ± 9.2^b^	142.2 ± 10.5^b^

The vehicle (distilled water) used to solve the tested medications was given at the same volume. Values (mean ± SD) were obtained from each group of 8 animals in each group after 12 weeks of the experimental period. ^a^ *P* < .05 and ^b^ *P* < .01 compared to the values of vehicle-treated HFD-fed rats in each group, respectively.

**Table 2:** Changes in the plasma lipids, hepatic lipids, atherogenic index (AI), and coronary artery index (CRI) in HFD-fed rats receiving 12-weeks treatment with VBRB or fenofibrate.

Groups	Plasma lipids (mg dL^−1^)					AI	CRI	Hepatic lipids (*μ*mol g^−1^ liver)	
	TC	TG	LDL-C	HDL-C	FFAs			TC	TG
RCD-fed									
Vehicle-treated	72.9 ± 5.8^b^	56.8 ± 6.6^b^	31.8 ± 3.2^b^	46.9 ± 3.4^b^	28.2 ± 2.8^b^	0.7 ± 0.3^b^	1.6 ± 0.4^b^	19.3 ± 2.7^b^	24.8 ± 6.2^b^
HFD-fed									
Vehicle-treated	130.2 ± 12.3	124.5 ± 6.3	120.2 ± 4.2	27.8 ± 3.8	61.9 ± 5.3	4.3 ± 0.2	4.7 ± 0.3	40.2 ± 3.1	54.4 ± 8.4
VBRB (g kg^−1^ per day)									
0.3	116.7 ± 10.3	110.9 ± 6.8^a^	92.5 ± 5.7^a^	32.1 ± 4.2^a^	55.3 ± 4.6	2.9 ± 0.2^a^	3.6 ± 0.3^a^	33.9 ± 3.5	48.6 ± 7.9
0.5	99.6 ± 8.3^a^	95.0 ± 5.2^a^	74.3 ± 5.1^b^	39.1 ± 2.9^a^	47.1 ± 3.8^a^	1.9 ± 0.1^b^	2.5 ± 0.3^b^	31.7 ± 4.1^a^	40.8 ± 7.1
1.0	89.1 ± 7.9^a^	80.3 ± 4.8^b^	68.1 ± 4.5^b^	43.9 ± 3.2^b^	38.7 ± 3.2^b^	1.5 ± 0.1^b^	2.0 ± 0.3^b^	26.9 ± 3.8^a^	35.2 ± 6.5^a^
Fenofibrate (100 mg kg^−1^ per day)	80.2 ± 8.0^b^	65.6 ± 4.7^b^	48.4 ± 4.3^b^	45.9 ± 4.4^b^	33.2 ± 4.1^b^	1.0 ± 0.2^b^	1.7 ± 0.4^b^	22.4 ± 4.5^b^	30.6 ± 7.8^b^

The vehicle (distilled water) used to solve the tested medications was given at the same volume. Values (mean ± SD) were obtained from each group of 8 animals in each group after 12 weeks of the experimental period. ^a^ *P* < .05 and ^b^ *P* < .01 compared to the values of vehicle-treated HFD-fed rats in each group, respectively.

**Table 3:** Results of histopathological findings in livers and epididymal adipocytes of HFD-fed rats receiving 12-weeks treatment with VBRB or fenofibrate.

Groups	Hepatic steatosis (0–4)	Average sizes of epididymal adipocytes (*μ*m^2^)
RCD-fed		
Vehicle	0^b^	3160.5 ± 230.3^b^
HFD-fed		
Vehicle	3.28 ± 0.56	5502.3 ± 210.9
VBRB (g kg^−1^ per day)		
0.3	2.64 ± 0.61	4480.2 ± 156.2^a^
0.5	2.58 ± 0.74	4072.8 ± 181.3^a^
1.0	2.23 ± 0.53	3501.2 ± 148.2^b^
Fenofibrate (100 mg kg^−1^ per day)	2.12 ± 0.39^a^	3226.5 ± 174.6^b^

The vehicle (distilled water) used to solve the tested medications was given at the same volume. Values (mean ± SD) were obtained from each group of 5 animals in each group during after 12 weeks of the experimental period. The size and number of epididymal adipocytes in a fixed area (1,000,000 *μ*m^2^) were measured. ^a^ *P* < .05 and ^b^ *P* < .01 compared to the values of vehicle-treated HFD-fed rats in each group, respectively.
